# Clinical practice recommendations for the treatment of Alport syndrome: a statement of the Alport Syndrome Research Collaborative

**DOI:** 10.1007/s00467-012-2138-4

**Published:** 2012-03-30

**Authors:** Clifford E. Kashtan, Jie Ding, Martin Gregory, Oliver Gross, Laurence Heidet, Bertrand Knebelmann, Michelle Rheault, Christoph Licht

**Affiliations:** 1 Department of Pediatrics, Division of Pediatric Nephrology, University of Minnesota Medical School, Minneapolis, MN USA; 2 Pediatric Department, Peking University First Hospital, Beijing, People’s Republic of China; 3 Division of Nephrology, University of Utah Health Sciences Center, Salt Lake City, UT USA; 4 Department of Nephrology and Rheumatology, University Medicine Goettingen, Goettingen, Germany; 5 Centre de référence pour les Maladies Rénales Héréditaires de l’Enfant et de l’Adulte (MARHEA) and Service de Néphrologie Pédiatrique, Hôpital Necker-Enfants malades, Paris, France; 6 The Hospital for Sick Children, Division of Nephrology, Toronto, Canada

**Keywords:** Alport syndrome, Proteinuria, Angiotensin-converting enzyme inhibitor, Angiotensin receptor blocker, Aldosterone inhibitor

## Abstract

We present clinical practice recommendations for the treatment of children with Alport syndrome who are not enrolled in clinical trials. Our goal is to promote early initiation of a standard therapeutic approach that will facilitate assessment of the safety and efficacy of the protocol. The treatment protocol is based on the reduction of proteinuria, intraglomerular pressure, and renal fibrosis via interference with the renin–angiotensin–aldosterone system.

## Introduction

Investigation of Alport syndrome (AS) has elucidated the roles of type IV collagen in basement membranes and described the consequences of type IV collagen mutations for renal, cochlear, and ocular structure and function. Research studies employing animal models of AS have suggested interventions that may delay or reverse the renal effects of type IV collagen mutations, but these approaches have yet to be prospectively examined in human subjects with the disease. While we wait for clinical trials to be organized, funded, and carried to completion, children and adults with AS continue to progress towards end-stage renal disease (ESRD). What can nephrologists offer in the meantime, and what can we learn from clinical practice about the efficacy and safety of common treatment approaches?

The Alport Syndrome Research Collaborative, currently comprising investigators in Canada, China, France, Germany, and the United States, has developed clinical practice recommendations aimed at standardizing therapy for people with AS who are not enrolled in clinical trials. It is our hope that adoption of a consistent approach to therapy will allow pooling and analysis of data about treatment responses and efficacy.

### Disclaimer

These are general clinical practice recommendations for off-label therapy with inhibitors of the renin–angiotensin–aldosterone system (RAAS) and may not be appropriate for all children with AS. It is expected that practitioners will carefully consider a child’s baseline renal function in applying these guidelines, will appropriately monitor for adverse effects of therapy, and will adjust or discontinue therapy as needed.

### Natural history of untreated Alport syndrome

#### Genetics and genotype-phenotype correlations

Alport syndrome can be transmitted as an X-linked, autosomal recessive or autosomal dominant disorder. About 80% of individuals with AS have X-linked disease (XLAS) due to mutations in the *COL4A5* gene. Males with XLAS progress inexorably to ESRD, with ESRD risks of 50% by age 25, 90% by age 40, and nearly 100% by age 60 [1]. Age at ESRD is strongly correlated with *COL4A5* genotype in males with XLAS [1–3]; risk of ESRD by age 30 is 90% for deletions and nonsense mutations of *COL4A5*, 70% for splicing mutations, and 50% for missense mutations. The known strong genotype-phenotype correlation provides a rationale for using *COL4A5* genotype data to guide the timing and intensity of intervention. In most families with XLAS, age at ESRD is fairly similar among affected males. In the absence of *COL4A5* genotype data, timing of ESRD can be predicted for a young affected male on the basis of ESRD timing in older affected male relatives.

The effects of *COL4A5* genotype on age at ESRD are not observed in females with XLAS, likely due to the overwhelming influence of X-inactivation [4]. In XLAS females, the timing and intensity of intervention should be guided by risk factors for progression to ESRD: proteinuria, gross hematuria, and hearing loss [5, 6].

Autosomal recessive AS (ARAS) accounts for about 15% of individuals with the disease and arises from mutations in both alleles of either *COL4A3* or *COL4A4*. Genotype-phenotype data for ARAS is relatively sparse. In general, individuals with ARAS carry a high risk of ESRD by age 30. Only about 5% of individuals with AS have autosomal dominant disease (ADAS). As ADAS tends to progress at a relatively slow velocity [7], there is less urgency to consider initiation of intervention in childhood.

#### Clinicopathological correlations

In general, AS is characterized by genetically determined dysfunction of the glomerular filter, mainly caused by mutations in the collagens assembling the glomerular basement membrane (GBM). In consequence, the earliest sign of GBM filter dysfunction is hematuria followed by albuminuria and subsequent nonselective proteinuria in increasing magnitude. Ultimately, not the GBM damage per se but the pro-inflammatory and pro-fibrotic consequences both in the tubulointerstitium and in the glomeruli resulting from progressive proteinuria, eventually lead to the development of ESRD [8].

Overt proteinuria is typically absent in infant males with XLAS. Age at identification of overt proteinuria shows interfamilial variability and ranges from early childhood to adolescence. In dogs with XLAS, a period of microalbuminuria precedes the development of overt proteinuria and quantitative increases in interstitial volume due to tubular atrophy and fibrosis [9]. Dogs with XLAS exhibit increased proximal tubular epithelial cell uptake of albumin, a process that has been linked to cellular injury [9]. Preliminary data from the Alport Syndrome Treatments and Outcomes Registry (ASTOR) indicates that boys with AS also exhibit a transitional stage of microalbuminuria before overt proteinuria becomes established (manuscript in preparation). It has yet to be demonstrated that suppression of microalbuminuria has anti-fibrotic effects in AS, although this is a reasonable hypothesis.

Measurements of cortical interstitial volumes in AS males have shown that interstitial fibrosis is unusual before age 10 [10]. Cortical interstitial volumes become abnormal in many AS males during the second decade of life, and are inversely correlated with glomerular filtration rates [10]. These observations suggest that, (1) as in other chronic glomerulopathies, interstitial fibrosis is a significant contributor to loss of renal function, and (2) prevention of interstitial fibrosis in AS males may require intervention during childhood.

### Therapeutic studies

Several therapies improve outcomes in animal models of AS, including angiotensin-converting enzyme (ACE) inhibition [11, 12], AT1-receptor blockade (ARB) [11, 12], inhibitors of TGF-β1, matrix metalloproteinases, vasopeptidase A or HMG-CoA reductase [13–16]; chemokine receptor 1 blockade [17], BMP-7 [18], stem cells [19–22], and irradiation [23]. However, none of these approaches has been prospectively studied in human AS populations.

In ARAS mice, initiation of ACE inhibitor therapy before onset of proteinuria suppressed proteinuria and azotemia and doubled length of survival [11], a therapeutic benefit that has yet to be exceeded by any other intervention. A smaller but still significant improvement in outcome was achieved when ACE inhibition was started after onset of proteinuria [11]. ACE inhibitor therapy begun before onset of proteinuria lengthened survival in canine XLAS [24].

Retrospective analysis of registry data strongly suggests that ACE inhibition delays ESRD and improves life expectancy in AS patients in a time-dependent manner [25]. This observation highlights the importance of early and accurate diagnosis of AS, including specific genotyping whenever feasible (for information on diagnostic methods in AS, please see www.genereviews.org). Besides the beneficial effects of RAAS blockade on outcomes in experimental and human AS, there are additional compelling reasons to recommend RAAS blockade in AS patients until a superior therapy is identified. First, ACE inhibitor therapy at doses that achieve suppression of proteinuria has been used with a high degree of safety in children with chronic kidney disease [26, 27]. Further, these agents are widely available and relatively inexpensive, making this therapy accessible to AS patients worldwide.

### Clinical trials

We recognize that a randomized clinical trial would be the best way to evaluate the efficacy of ACE inhibition or any other intervention in children with AS. We also recognize that widespread adoption of our recommendations may reduce the pool of children who are eligible for clinical trials of potential therapies for AS. Nevertheless, we encourage eligible subjects and their families to consider participation in clinical trials wherever and whenever possible. For example, by the time this document is published, a clinical trial of ACE inhibition in AS will be recruiting subjects residing in Germany, or willing and able to travel to Germany (contact: studie@alport.de; for information and to download flyer see www.alport.de/EARLY_PRO-TECT). The goal of this trial is to clarify if an early start of therapy (in patients with isolated hematuria or microalbuminuria) delays renal failure even more effectively than later onset of therapy (in proteinuric patients) and above all if therapy at early stages of AS is safe.

For those subjects unable to take part in clinical trials, we believe that valuable efficacy and safety data can be collected if these subjects are treated according to a standardized protocol. Currently, many children with AS are receiving ACE inhibitor therapy in a non-standardized fashion, and information about treatment responses is not being centrally collected or analyzed. We believe that this represents a wasted opportunity.

### Treatment recommendations

Retrospective registry data strongly suggests that in subjects with AS ACE inhibitor therapy initiated once proteinuria has developed but while glomerular filtration rate is well preserved delays ESRD [25]. Similarly, in mice and dogs with AS, interventions that delay the onset of proteinuria or reduce established proteinuria prolong renal survival [11, 24]. Based on these observations, and on the general consensus that suppression of intraglomerular pressure and proteinuria is an important component of the management of chronic glomerular diseases, treatment of proteinuric AS patients with medications that diminish proteinuria is recommended. Proteinuria in children is defined as urine protein-creatinine ratio persistently greater than 0.2 mg/mg in children over 2 years of age, or urinary protein excretion greater than 4 mg/m^2^/h in a timed collection [28, 29].

Cortical interstitial volume fraction (VvI/C) is a measure of interstitial fibrosis and tubular atrophy [30]. There is a strong inverse correlation between VvI/C and creatinine clearance in boys with AS [10]. In these boys, VvI/C is typically within the normal range during the first decade of life, when creatinine clearance is also normal, but often begins to increase during adolescence concurrent with declining creatinine clearance. We presume that *COL4A5* mutations associated with relatively rapid progression to ESRD, such as deletions, nonsense, and splicing mutations, result in earlier onset and more aggressive development of interstitial fibrosis and tubular atrophy. In dogs and mice with AS, the onset of proteinuria precedes measurable increases in interstitial fibrosis. In light of these observations, we recommend that in boys with AS who have deletion, nonsense or splicing mutations, or who have a family history of ESRD before age 30, monitoring of urine protein excretion should begin early in life and that an aggressive approach to initiating and escalating proteinuria-suppressing therapies should be followed.

**Fig. 1:**
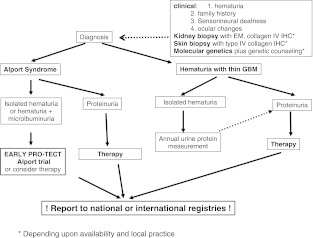
Algorithm for identifying children with familial hematuria (Alport syndrome or hematuria with thin glomerular basement membranes) who are candidates for intervention.* IHC* immunohistochemistry; *EM* electron microscopy; *GBM* glomerular basement membrane *Depending upon availability and local practice

Based on these principles, we make the following recommendations aimed at preventing renal tubular epithelial cell injury and suppressing fibrogenic processes in the renal interstitium (see Fig. 1):

1. Monitoring for microalbuminuria and proteinuria should be initiated by age 1 year in at risk children, or as soon as a diagnosis of Alport syndrome is established, and repeated at least annually.
2. Affected individuals with overt proteinuria (urine protein-creatinine ratio persistently greater than 0.2 mg/mg, or urinary protein excretion greater than 4 mg/m^2^/h in a timed collection) should receive treatment.
3. Treatment should be considered in affected boys with microalbuminuria in whom the risk of ESRD by age 30 is high, such as those with *COL4A5* deletions, nonsense or splicing mutations, or a history of ESRD before age 30 in affected male relatives (Table 1). We recognize that access to molecular genetic testing for Alport syndrome, and coverage by insurers, is variable. We recommend that the Alport genotype be determined whenever feasible, to facilitate identification of those at high risk of ESRD by age 30.

**Table 1:** Recommendations for intervention based on urinary findings and anticipated disease course

	Family history of early ESRD (< 30 years) or severe *COL4A5* mutation^a^		Family history of late ESRD (> 30 years) or less severe *COL4A5* mutation^b^	
	Male	Female	Male	Female
Hematuria	Intervention prior to onset of microalbuminuria is not recommended at this time	No	No	No
Hematuria + microalbuminuria	Consider intervention	Consider intervention	No	No
Hematuria + proteinuria	Yes	Yes	Yes	Yes

^a^Deletion, nonsense, or splicing mutation

^b^Missense mutation

*ESRD* end stage renal disease

#### Target

The optimal target for lowering of urine protein levels is uncertain. Our recommendations are based upon an arbitrary goal of a urine protein:creatinine ratio of less than 0.5 mg/mg if the baseline value is greater than 1.0 mg/mg, or a 50% reduction if the baseline value is greater than 0.2 but less than 1.0. When therapy is initiated in subjects with microalbuminuria, we recommend a target microalbumin:creatinine ratio of less than 50–100 mg/g creatinine.

Proteinuria may persist at levels that exceed these targets, despite maximum dosing of first- and second-line agents. In these cases, we recommend continuing therapy, with adjustment of dosing as indicated by growth and by renal function.

#### Agents

##### First line

We chose angiotensin-converting enzyme (ACE) inhibition as first-line therapy for several reasons. First, ACE inhibition is the choice of most nephrologists for initial non-immunologic therapy of proteinuric glomerular disease. Consequently, practitioners have extensive experience with dosing these agents and are familiar with their adverse effects. ACE inhibitors are widely available and relatively inexpensive. The Evaluation Study of Congestive Heart Failure and Pulmonary Artery Catheterization Effectiveness (ESCAPE) trial demonstrated that ACE inhibition with ramipril is associated with very low frequencies of adverse events in children with chronic kidney disease and at least transient reductions in proteinuria [26, 27]. Because of the ESCAPE experience, we chose ramipril as the reference ACE inhibitor, and suggest equivalent doses of other ACE inhibitors in Table 2. Finally, ramipril therapy started before or after onset of proteinuria significantly prolonged survival in mice with autosomal recessive Alport syndrome, and its effects were superior to those of candesartan [11, 12].

**Table 2:** First-line therapy (angiotensin-converting enzyme inhibitor)

Agent	Dose
Ramipril	Starting dose of 1 to 2 mg/m^2^/day; increase by 1 to 2 mg/m^2^/day every 3 months until target UPC or adverse effect is attained; maximum dose 6 mg/m^2^/day
Enalapril	2 × Ramipril dose (2 to 4 mg/m^2^/day)
Lisinopril	4 × Ramipril dose (4 to 8 mg/m^2^/day)
Benazepril	
Fosinopril	
Quinapril	
Cilazapril	1 × Ramipril dose (1 to 2 mg/m^2^/day)
Perinopril	
Trandolapril	0.5 × Ramipril dose (0.5 to 1 mg/m^2^/day)

In the ESCAPE trial, reduction in proteinuria and preservation of glomerular filtration rate were correlated with ramipril’s antihypertensive effect [26, 27]. Many of the Alport subjects in whom therapy is initiated when they have developed microalbuminuria or mild proteinuria will be normotensive. If the renoprotective properties of ACE inhibitors are primarily due to their antihypertensive effects, the impact on Alport subjects may be insignificant. However, ramipril delays proteinuria and prolongs survival in normotensive autosomal recessive Alport syndrome mice, indicating that other effects of ACE inhibition are important in this model [11, 12].

##### Second line

We propose two alternatives for second-line therapy, angiotensin receptor blockade (ARB) and aldosterone inhibition. For those nephrologists with experience combining an ACE inhibitor with an ARB, ARB may be the more comfortable, familiar approach. We suggest losartan as the reference ARB, based on published experience in children with chronic kidney disease [31]. In a recent study of 30 proteinuric children with AS, losartan was found to reduce proteinuria to a greater extent than placebo or amlodipine [32]. We recommend a relatively low starting dose, given that the ARB will be added to ACE inhibition (Table 3). If an ARB is used instead of an ACE inhibitor, for example because a patient tolerates ACE inhibition poorly, a higher starting dose for the ARB would be appropriate.

**Table 3:** Second-line therapy (angiotensin receptor blocker)

Agent	Dose
Losartan	12.5 mg/m^2^/day; double dose every 3 month until target UPC or adverse effect is attained; maximum dose 50 mg/m^2^/day
Candesartan	0.2 × Losartan dose (6.25 mg/m^2^/day)
Irbesartan	3 × Losartan dose (37.5 mg/m^2^/day)
Telmisartan	0.8 × Losartan dose (10 mg/m^2^/day)
Valsartan	1.5 × Losartan dose (18.75 mg/m^2^/day)
Epresartan	12 × Losartan dose (150 mg/m^2^/day)

There are a limited number of published reports regarding the use of combination therapy with an ACE inhibitor and an ARB in children with chronic proteinuric renal diseases. Ten children with chronic kidney disease and persistent proteinuria, despite maximal doses of an ACE inhibitor, exhibited a sustained reduction in proteinuria after addition of losartan, with no significant changes in blood pressure or glomerular filtration rate [33]. In another small study of ten children with chronic kidney disease and proteinuria that employed a cross-over design, combination therapy with an ACE inhibitor and an ARB reduced proteinuria to a significantly greater extent than either agent alone [34].

In a small group of patients with AS, combined therapy with an ACE inhibitor and spironolactone suppressed proteinuria to a greater extent than the combination of an ACE inhibitor with an ARB [35]. Hyperkalemia was not encountered in this small group of patients. Increased aldosterone levels (aldosterone escape) may contribute to persistent proteinuria in Alport patients receiving ACE inhibitor therapy [36]. Aldosterone inhibition could be used as the initial second-line agent, or as an alternative to ineffective ARB therapy (Table 4).

**Table 4:** Second-line therapy (aldosterone antagonist)

Agent	Dose
Spironolactone	25 mg daily for subjects 10–20 years of age; consider lower starting dose in children less than 10 years of age

#### Potential adverse effects of therapy

Potential adverse effects of the therapeutic approach described above include orthostatic hypotension, fetopathy in ovulating females, hyperkalemia, reversible decline in glomerular filtration rate, and gynecomastia. In the ESCAPE trial, only three of 352 children who received ramipril for at least 6 months required discontinuation of therapy because of orthostatic hypotension or hyperkalemia [24]. ACE inhibitors and ARBs should be used with caution in ovulating females in order to avoid fetal injury.

In the ONgoing Telmisartan Alone and in Combination with Ramipril Global Endpoint Trial (ONTARGET) study, the combination of ramipril and telmisartan resulted in increased risk of adverse renal outcomes (doubling of serum creatinine and requirement for dialysis), hypotensive symptoms, syncope, and hyperkalemia, compared to ramipril monotherapy [37]. Subjects who received combination therapy also had an increased risk of mortality, although not statistically significant [37]. The relevance of the results of the ONTARGET study, in which study subjects had a mean age of 66 years and were at high risk for cardiovascular events due to vascular disease or diabetes, to children and young adults with Alport syndrome is unclear.

Chronic administration of spironolactone is associated with an increased incidence of serum potassium greater than 5.5 mEq/l (about 3% of subjects participating in clinical trials), gynecomastia (about 5% of patients), and acute renal insufficiency (1–5% of subjects) [38]. We recommend regular monitoring of serum potassium and creatinine levels in treated Alport patients, especially those receiving combination therapy.

#### Reporting of treatment experience

We are in the process of developing an online reporting tool that will allow practitioners to upload de-identified information regarding treatment responses to a secure site. Once the tool is in place, we will assist interested practitioners in obtaining local IRB approval for participation. We will use listservs and blast e-mails from the Alport Syndrome Treatments and Outcomes Registry to announce the availability of the site. In the meantime, we encourage practitioners to share treatment experiences via e-mail with Dr. Kashtan.
